# Causal relationship between inflammatory proteins and glioblastoma: a two-sample bi‑directional mendelian randomization study

**DOI:** 10.3389/fgene.2024.1391921

**Published:** 2024-05-09

**Authors:** Xiang Lin, Wei Gao, Chen Huang, Minghua Wu, Xiaoling She

**Affiliations:** ^1^ Department of Pathology, The Second Xiangya Hospital, Central South University Changsha, Hunan, China; ^2^ Key Laboratory of Carcinogenesis and Cancer Invasion of Ministry of Education, Cancer Research Institute, Central South University, Changsha, Hunan, China; ^3^ NHC Key Laboratory of Carcinogenesis, Xiangya Hospital, Central South University, Changsha, Hunan, China; ^4^ Center of Basic Medical Research, Institute of Medical Innovation and Research, Peking University Third Hospital, Beijing, China

**Keywords:** Mendelian randomization, glioblastoma, neuroinflammation, inflammatory proteins, risk

## Abstract

**Background:** Observational studies have indicated a potential correlation between glioblastoma and circulating inflammatory proteins. Further investigation is required to establish a causal relationship between these two factors.

**Methods:** We performed a Mendelian randomization (MR) analysis using genome-wide association study (GWAS) summary of 91 circulating inflammation-related proteins (N = 14,824) to assess their causal impact on glioblastoma. The GWAS summary data for glioblastoma included 243 cases and 287,137 controls. The inverse variance weighted (IVW) method was used as the primary analytical method to assess causality. Four additional MR methods [simple mode, MR-Egger, weighted median, and weighted mode] were used to supplement the IVW results. Furthermore, several sensitivity analyses were performed to assess heterogeneity, horizontal pleiotropy, and stability. Reverse MR analysis was also performed. glioblastoma transcriptomic data from The Cancer Genome Atlas (TCGA) were analyzed to validate the findings obtained through MR, while pathway and functional enrichment analyses were conducted to predict the potential underlying mechanisms.

**Results:** Our findings from employing the inverse variance weighted method in our forward MR analysis provide robust evidence supporting a potential association between glioblastoma and elevated levels of Cystatin D, as well as decreased levels of fibroblast growth factor 21 (FGF21) in the circulation. Moreover, our reverse MR analysis revealed that glioblastoma may contribute to increased concentrations of C-X-C motif chemokine 9 (CXCL9) and Interleukin-33 (IL-33) in the bloodstream. Transcriptomic analysis showed that FGF21 expression was inversely associated with the risk of developing glioblastoma, whereas an increased risk was linked to elevated levels of CXCL9 and IL-33. Pathway and functional enrichment analyses suggested that Cystatin D might exert its effects on glioblastoma through intracellular protein transport, whereas FGF21 might affect glioblastoma via glucose response mechanisms.

**Conclusion:** These results indicate that FGF21 is a significant factor in glioblastoma susceptibility. Glioblastoma also affects the expression of inflammatory proteins such as C-X-C motif chemokine 9 and Interleukin-33, providing new insights into the mechanisms of glioblastoma genesis and clinical research.

## 1 Introduction

Glioblastoma is an extremely aggressive and invariably fatal form of brain cancer that poses significant treatment challenges owing to its resistance to current radiation and chemotherapy modalities (van Solinge et al., 2022). To overcome these therapeutic obstacles, extensive efforts have been made to comprehend the pathophysiological mechanisms underlying glioblastoma (Esemen et al., 2022). However, despite the exhaustive research conducted thus far, ionizing radiation remains the only well-established risk factor for this disease, while other potential predictors of glioblastoma development remain uncertain (Braganza et al., 2012).

Emerging research suggests that the aberrant activation of inflammatory responses plays a pivotal role in the progression and proliferation of glioblastoma (Chen et al., 2022). Previous studies have demonstrated significantly elevated levels of specific inflammatory markers, including interleukin-6 (IL-6), vascular endothelial growth factor (VEGF), and tumor necrosis factor-alpha (TNF-α), in the bloodstream of individuals diagnosed with glioblastoma compared to a control group comprising healthy individuals (Feng et al., 2019; Linhares et al., 2020). However, it remains uncertain whether these inflammatory proteins are intricately linked to the pathophysiology of glioblastoma or whether they have a causal relationship with the condition.

Mendelian randomization (MR) is an observational study that employs genetic variants as instrumental variables to estimate the causal impact of risk factors on health outcomes. Unlike conventional multivariate observational analyses, MR is less susceptible to confounding variables and measurement errors, thereby mitigating the bias caused by reverse causality. Consequently, MR has emerged as a reliable approach for obtaining robust estimates of the causal influence of various risk factors on health outcomes, often yielding results similar to those of randomized controlled trials (RCTs), if available (Skrivankova et al., 2021; Ye et al., 2024).

However, to date, no study has assessed the potential of MR to investigate the association between inflammatory proteins and glioblastoma. Therefore, this study aimed to employ an MR framework and two-sample methodology to explore a plausible causal relationship between inflammatory proteins and glioblastoma. The primary objective was to establish a theoretical foundation for understanding the intricate interplay between inflammatory proteins and glioblastoma development and progression.

## 2 Materials and methods

### 2.1 Mendelian randomization assumptions

**FIGURE 1:**
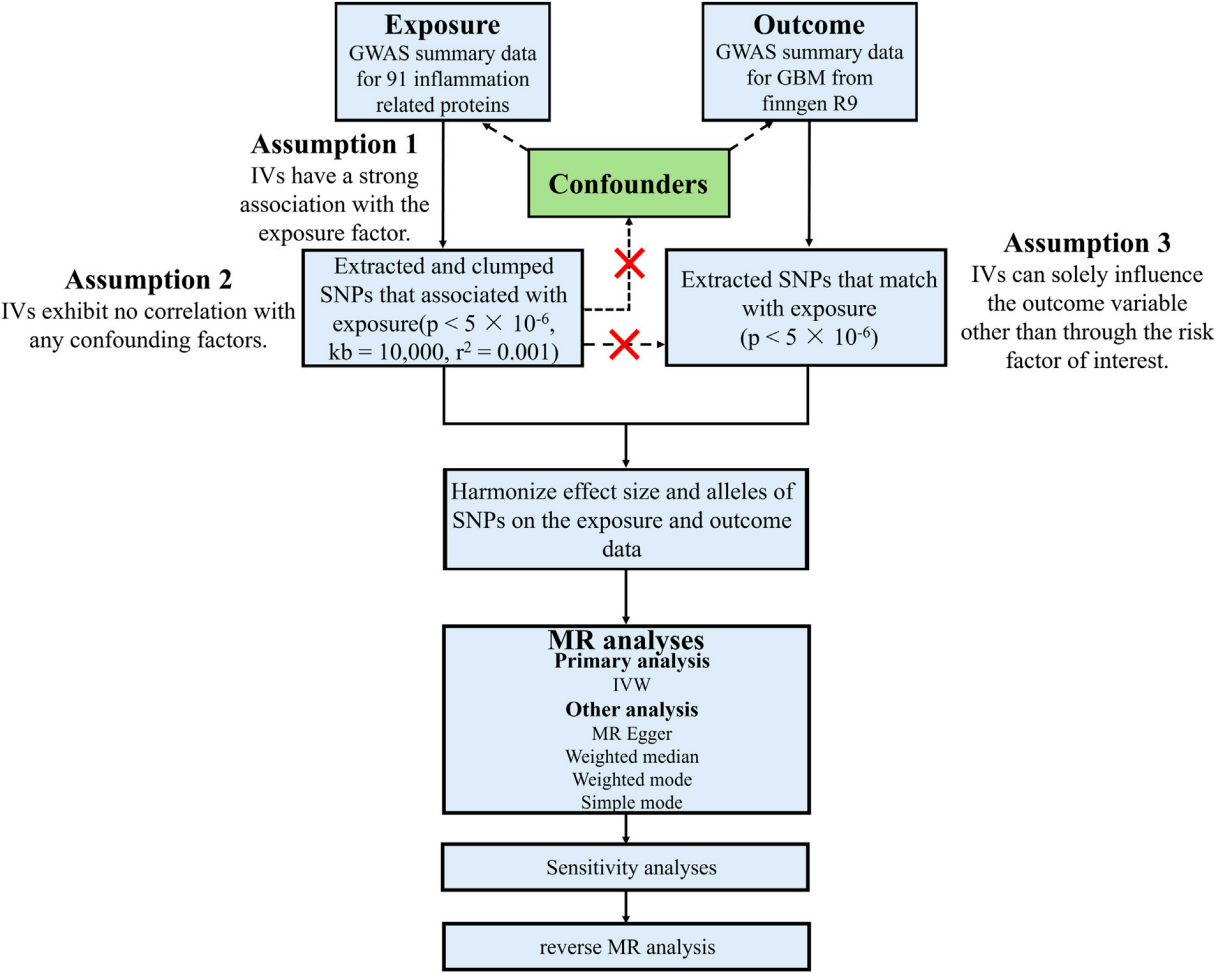
Study design and workflow of this study. GBM, Glioblastoma; GWAS, Genome-wide association studies; IVs, Instrumental variables; IVW, Inverse variance weighted; kb, Kilobase; MR, Mendelian randomization; *p*, *p*-value; *r* ^2^, Explained variance; SNP, Single-nucleotide polymorphism.

There are three fundamental assumptions underlying the analysis of Mendelian randomization (MR) (van Solinge et al., 2022): the instrumental variables must exhibit a strong association with the exposure factor (Esemen et al., 2022); the instrumental variables should not be correlated with any confounding factors related to the relationship between exposure and outcome (Braganza et al., 2012); The instrumental variables can only influence the outcome variable through their impact on the exposure factor. In this study, two genome-wide association studies (GWASs) were employed to identify genetically significant single nucleotide polymorphisms (SNPs) associated with 91 inflammatory proteins and glioblastoma (Figure 1).

### 2.2 Exposure data

Data on circulating inflammatory proteins comes from a recent study that includes 11 cohorts, totaling 14,824 participants of European ancestry. Genome-wide genetic data for 91 plasma proteins were measured using the Olink Target-96 Inflammation panel (Zhao et al., 2023).

### 2.3 Outcome data

The MR analysis utilized two publicly accessible summarized GWAS datasets. The glioblastoma GWAS summary statistics were acquired from the FinnGen consortium (Release 9, https://www.finngen.fi/en), encompassing 243 instances and 287,137 controls for brain glioblastoma (all cancers excluded) (Kurki et al., 2023).

### 2.4 Instrumental variable selection

For inflammatory proteins, we firstly applied a significance threshold (*p* < 5 × 10^−6^) and clumped these SNPs (kb = 10,000, *r* ^2^ = 0.001) in the context of forward MR analysis. Palindromic SNPs were discarded. GWAS data for glioma were adhered to the stringent significance threshold (*p* < 5 × 10^−6^) and linkage disequilibrium criteria (kb = 10,000, *r* ^2^ = 0.001) in reverse MR analysis. To evaluate the potential presence of weak instrument bias, the proportion of variance in exposure was calculated using the *R* ^2^ value of each SNP, and the instrument strength was estimated using the F-statistic. F-value exceeds 20 indicating the absence of such bias. To see how these data correlate with other clinical databases, we validated the results of the data against The Cancer Genome Atlas (TCGA) glioblastoma database platform for the HG—U133A using the GlioVis tool (http://gliovis.bioinfo.cnio.es/) and Gepia2 tool (http://gepia2.cancer-pku.cn/) (Bowman et al., 2016; Tang et al., 2019).

### 2.5 Gene annotation

Gene Mapping were applied in FUMA to identify genes that are associated with the SNPs, which were included in the MR analysis (https://fuma.ctglab.nl/) (Watanabe et al., 2017).

### 2.6 Pathway and functional enrichment analyses

Enrichment analyses were carried out on Metascape (https://metascape.org/gp/index.html#/main/step1) (Zhou et al., 2019).

### 2.7 Statistical methods

All statistical analyses for the MR study were conducted using R software (version 4.3.2) and the “TwoSampleMR” R package.

We employed five different MR techniques to investigate the potential causal link between inflammatory proteins and glioblastoma. The primary method used was inverse variance weighted (IVW), supplemented by other methods such as MR‒Egger and Weighted Median for further analysis. A *p*-value less than 0.05 indicated a significant causal effect, indicating an increased risk of exposure leading to the outcome. We assessed the relationship between circulating inflammatory proteins and glioblastoma using odds ratios (OR). Heterogeneity was evaluated using Cochran’s Q statistic in IVW methods, with heterogeneity considered absent when *p* > 0.05. In cases of heterogeneity, random-effects models were utilized to exclude or estimate SNP effects. If no significant heterogeneity was observed (*p* < 0.05), a fixed-effect model was adopted. Pleiotropy was examined through *p*-values derived from MR‒Egger regression and MR-PRESSO; a *p*-value exceeding 0.05 suggested no potential pleiotropic effects existed. Additionally, we conducted sensitivity analyses via a “leave-one-out” approach to demonstrate that individual SNPs did not unduly influence the causal relationship between exposure and outcome.

## 3 Results

### 3.1 Exploration of the causal effect of inflammatory proteins on GBM risk

Considering the limited extent of genetic variation, a restricted number of single nucleotide polymorphisms (SNPs), and their relatively moderate effect sizes, we conducted an MR analysis using a lenient *p*-value threshold of 5 × 10^−6^ to ensure sufficient SNP coverage for subsequent MR analysis (Supplementary Tables S1, S2). The primary results of forward MR analyses of the 91 inflammatory proteins are shown in Supplementary Table S3.

**FIGURE 2:**
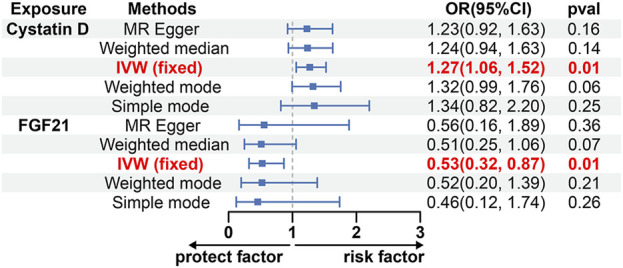
The causal effect of Cystatin D and FGF21 on GBM estimated using 5 MR methods. CI, Confidence interval; FGF21, Fibroblast growth factor 21; IVW, Inverse variance weighted; MR, Mendelian randomization; OR, Odds ratios; *p*, *p*-value.

Among the various factors examined, Cystatin D exhibited a positive correlation with the risk of glioblastoma (Odds ratio [OR], 1.27; 95% confidence interval [CI]: 1.06–1.52; *p* = 0.01) when utilizing inverse variance weighted (IVW) methods (Figure 2). This analysis showed no significant heterogeneity (MREgger Q = 24.27, Q *p*-value = 0.76) or horizontal pleiotropy (*p* Egger intercept = 0.75, *p* MR Presso = 0.98) (Supplementary Table S4). Additionally, our findings indicated that fibroblast growth factor 21 (FGF21) was associated with a decreased likelihood of developing glioblastoma according to the IVW approach (OR, 0.53; 95% CI: 0.32–0.87; *p* = 0.01), with the analysis showing no significant heterogeneity (MR Egger Q = 9.59, Q *p*-value = 0.65) and no horizontal pleiotropy (P Egger Intercept = 0.92, P MR Presso = 0.70) (Figure 2; Supplementary Table S4). However, despite similar associations observed in other analyses, no causal effects of Cystatin D and FGF21 on glioblastoma were identified (*p* > 0.05) (Figure 2). Moreover, we conducted sensitivity analysis by excluding one SNP locus at a time to assess the impact of each SNP on the overall causal relationship. No significant deviations were observed in the observed causal relationship when systematically removing individual SNPs and reperforming the MR analysis (Supplementary Figures S1, S2). There was no evidence of a causal relationship between the other circulating inflammatory proteins and glioblastoma (Supplementary Table S3).

### 3.2 Exploration of the causal effect between GBM risk and inflammatory proteins

To explore the possibility of reverse causation, we conducted a comprehensive analysis of seven SNPs that exhibited robust and autonomous correlations with glioblastoma, employing a significance threshold of *p* < 5 × 10^−6^ (Supplementary Tables S5, S6).

**FIGURE 3:**
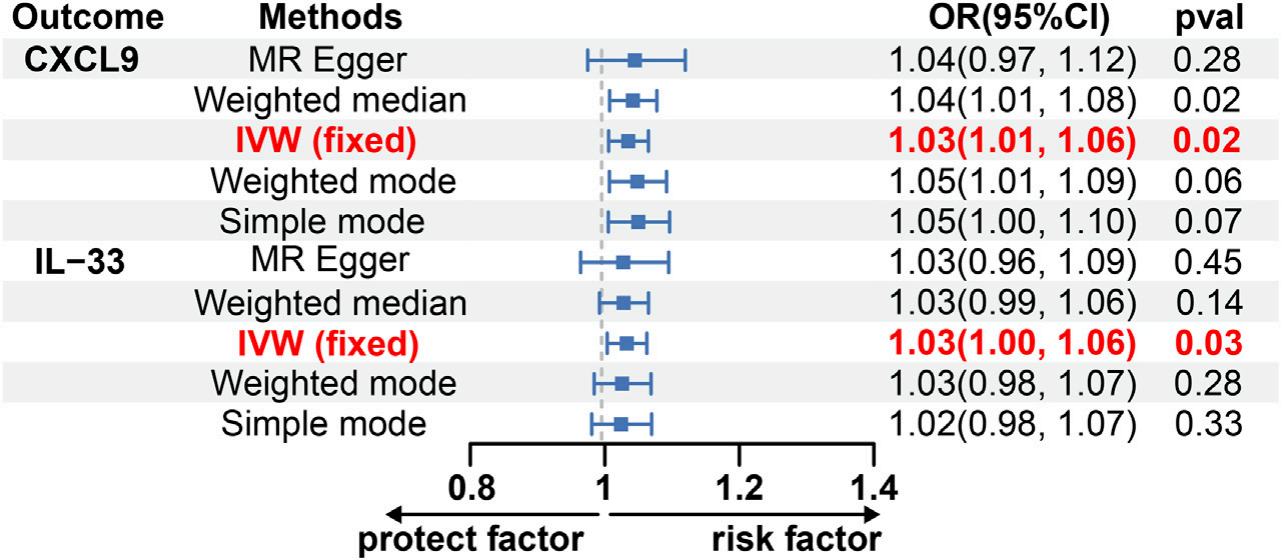
The causal effect of GBM on CXCL9 and IL-33 estimated using 5 MR methods. CXCL9, C-X-C motif chemokine 9; CI, Confidence interval; IL-33, Interleukin-33; IVW, Inverse variance weighted; MR, Mendelian randomization; OR, Odds ratios; *p*, *p*-value.

Using the IVW method, we discovered a possible correlation between an increased risk of glioblastoma and elevated levels of C-X-C motif chemokine 9 (CXCL9) and Interleukin-33 (IL-33) (OR, 1.03; 95% CI: 1.01, 1.06; *p* = 0.021; OR, 1.03; 95% CI: 1.03, 1.06; *p* = 0.030) (Figure 3; Supplementary Table S7). To validate the reliability of these findings, pleiotropy tests were performed on the included SNP loci, which yielded no evidence of horizontal pleiotropy [*p* Egger intercept (CXCL9) = 0.78, *p* MR presso (CXCL9) = 0.98; *p* Egger intercept (IL-33) = 0.87, *p* MR presso (IL-33) = 0.70; Supplementary Table S7]. Cochran’s Q test suggested no heterogeneity between IL-33 and CXCL9 [MR Egger Q (CXCL9) = 7.60, Q *p*-value  (CXCL9) = 0.17; MR Egger Q (IL-33) = 3.40, Q *p*-value (IL-33) = 0.64; Supplementary Table S7]. Sensitivity analysis using the leave-one-out approach yielded robust results (Supplementary Figures S3, S4). Notably, there was no evidence of a causal relationship between glioblastoma and Cystatin D or FGF21 levels (Supplementary Table S7).

### 3.3 Differential expression of inflammatory proteins between GBM and normal cells

To investigate the role of Cystatin D, FGF21, IL-33 and CXCL9 in glioblastoma, we analyzed the correlation between the expression of these genes and survival prognosis characteristics in patients with glioblastoma (Bowman et al., 2016). The results demonstrated a significant downregulation of FGF21 expression in glioblastoma tissues, whereas both CXCL9 and IL-33 were notably upregulated (Supplementary Figure S5). Additionally, increased FGF21 expression positively correlated with improved overall survival (OS), whereas increased levels of CXCL9 and IL-33 were associated with decreased OS in patients with glioblastoma, although the difference was not statistically significant (Supplementary Figure S5). Despite the observed upregulation of Cystatin D (CST5) expression in the glioblastoma tissues, no significant differences were detected. Furthermore, elevated CST5 expression did not affect the OS of glioblastoma patients (Supplementary Figures S5, S6). Collectively, these findings suggest that FGF21 expression is inversely associated with the risk of developing glioblastoma, whereas an increased risk is linked to elevated levels of CXCL9 and IL-33.

### 3.4 Functional annotation and gene set enrichment analyses

Using position-based localization in FUMA, we identified 39 genes related to Cystatin D and 15 genes related to FGF21 by mapping risk loci and independent GWAS SNPs (Supplementary Tables S8, S9) (Watanabe et al., 2017). Subsequent tissue-specific analyses confirmed the expression of these identified genes, specifically in brain tissue (Supplementary Figures S7, S8) (Consortium et al., 2020).

**FIGURE 4:**
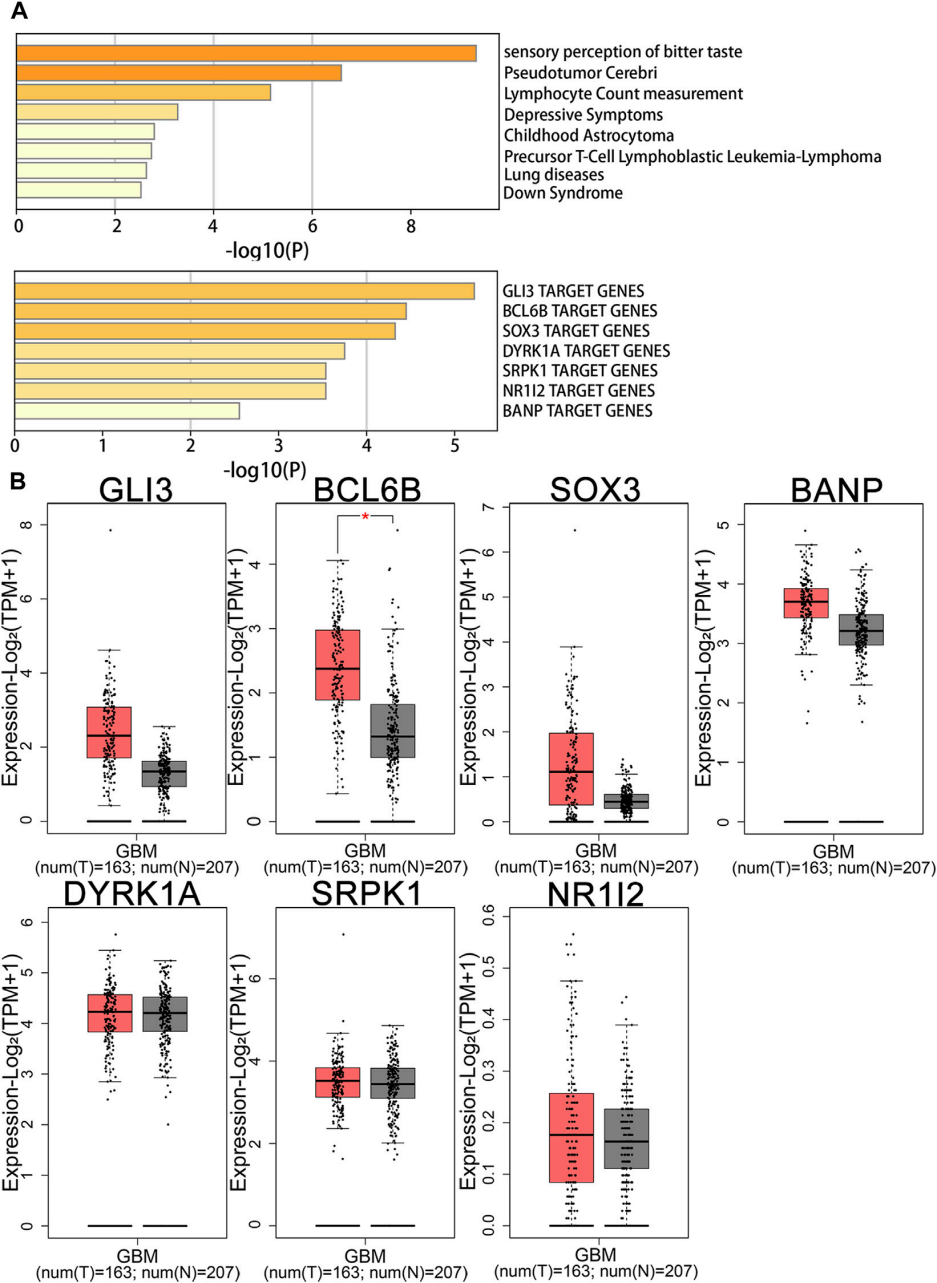
Enrichment and gene expression analysis of Cystatin D. **(A)** Summary of enrichment analysis in DisGeNET and transcription factor. **(B)** Gene expression analysis of transcription factor in GBM patients and normal people. BANP, BTG3 associated nuclear protein; BCL6B, B-cell CLL/lymphoma 6 member B protein; DYRK1A, Dual specificity tyrosine phosphorylation regulated kinase 1A; GBM, Glioblastoma; GLI3, GLI family zinc finger 3; N, Normal; NR1I2, Nuclear receptor subfamily 1 Group I member 2; SOX3, SRY-Box transcription factor 3; SRPK1, SRSF protein kinase 1; T, Tumor.

Enrichment analysis using DisGeNET revealed that genes related to Cystatin D expression in the brain were associated with childhood astrocytoma and pseudotumor cerebri (Figure 4), whereas genes related to FGF21 were linked to gout-related diseases (Supplementary Figure S9). Transcription factor enrichment analysis suggested that GLI Family Zinc Finger 3 (GLI3), B-cell CLL/lymphoma 6 member B protein (BCL6B), SRY-Box Transcription Factor 3 (SOX3), Dual Specificity Tyrosine Phosphorylation Regulated Kinase 1A (DYRK1A), SRSF Protein Kinase 1 (SRPK1), Nuclear Receptor Subfamily 1 Group I Member 2 (NR1I2), and BTG3 Associated Nuclear Protein (BANP) may serve as key regulators of Cystatin D-related genes (Figure 4). Among these, BCL6B was highly expressed in the tissues of patients with glioblastoma (Figure 4). Nuclear Receptor Subfamily 1 Group H Member 4 (NR1H4) and paired-like homeodomain 1 (PITX1) may be key regulators of FGF21-related genes (Supplementary Figure S10). Among them, PITX1 was highly expressed in glioblastoma patient tissues (Supplementary Figure S11) (Piñero et al., 2019; Tang et al., 2019; Zhou et al., 2019).

## 4 Discussion

Similar to other malignant cancers, substantial evidence supports the involvement of inflammation in the diagnosis, progression, and prognosis of glioblastoma (Zhao et al., 2021). A recent meta-analysis proposed that circulating interleukin 6 (IL-6) and C-Reactive Protein (CRP) could potentially serve as robust biomarkers for predicting poor prognosis in patients with glioma (Feng et al., 2019). However, limited research has been conducted on the association between inflammation and the incidence of glioma.

Herein, we present a comprehensive MR analysis to investigate the association between circulating inflammatory proteins and glioblastoma. Two-sample bidirectional MR was used to identify the circulating inflammatory proteins associated with glioblastoma. Additionally, transcriptome analysis was performed to validate the findings of MR analysis. Consequently, we conclude that elevated levels of circulating FGF21 are inversely correlated with the risk of developing glioblastoma.

FGF21 was discovered in 2000 as a novel member of the FGF family and is primarily expressed in the liver. Unlike other members of this family, FGF21 does not stimulate cell proliferation, but acts as a potent metabolic regulator (Lu et al., 2021). Its biological effects are mediated by binding with high affinity to klotho beta (KLB)-facilitated FGF receptor 1 (FGFR1), FGFR2, and FGFR3. For instance, when confronted with excessive fat accumulation, FGF21 promotes lipid oxidation to reduce hepatic fat content. Conversely, FGF21 deficiency is associated with an increased risk of liver, prostate, clear cell renal cell carcinoma, and breast cancer (Kim et al., 2022). Although undetectable in the nervous system, FGF21 can penetrate the blood-brain barrier and provide neuroprotection to the CNS (Sui and Chen, 2022). Despite limited research on FGF21 in glioblastoma, we conducted enrichment analysis for genes associated with FGF21 using gene clustering tools to explore their potential biological mechanisms in glioblastoma. Our findings indicated that FGF21-related genes were mainly enriched in response to carbohydrates (Supplementary Table S10). Altered tumor metabolism is a defining hallmark of glioblastoma; therefore, the regulation of abnormal cell metabolic states through FGF21 may reduce the risk of developing glioblastoma (Agnihotri and Zadeh, 2015). In addition, our transcriptomic analysis revealed the role of FGF21 in glioblastoma. We found that in patients with glioblastoma, the expression of FGF21 in tumor tissues was reduced, and these patients had longer overall survival. These findings provide new insights and highlight the potentially important role of FGF21 in the pathogenesis of glioblastoma.

Although we identified a positive association between Cystatin D and the risk of glioblastoma in our MR Analysis, the data of CST5 did not show a significant difference in our transcriptomic analysis. This difference can be attributed to various reasons. The effects of CST5 may not have been captured at the transcript level, or the effect size was too small to be detected in our sample size. Cystatin D, a predominant type-2 cysteine protease inhibitor in saliva, belongs to the cystatin superfamily of endogenous inhibitors that target endosomal/lysosomal cysteine proteases including cathepsins B, H, L and S. Studies have identified Cystatin D as a potential tumor suppressor gene in colon cancer due to its ability to antagonize the Wnt/β-catenin signaling pathway and repress c-MYC expression, thereby inhibiting cell proliferation. Furthermore, Cystatin D also suppresses epithelial-mesenchymal transition (Serrao et al., 2023; ρlvarez-Díaz et al., 2009). We performed a gene clustering analysis to conduct an enrichment analysis of the genes associated with CST5. Our findings suggested that genes related to CST5 were predominantly enriched in the intracellular protein transport pathway (Supplementary Table S10). Previous studies have demonstrated that glioblastoma cells release microvesicles containing mRNA, miRNAs, and angiogenic proteins, thereby facilitating glioblastoma growth (Skog et al., 2008). Despite the lack of significant findings for CST5 in our transcriptomic analysis, our MR results and enrichment analysis suggested that it might still play a role in the disease process.

Reverse MR and transcriptomic analyses in this study also revealed a potential correlation between glioblastoma, CXCL9, and IL-33. De Boeck et al. (2020) reported that glioma-derived IL-33 promotes glioma progression *in vivo* by modulating the cellular state of the tumor microenvironment. The initial characterization of the *in vivo* glioma secretome identified IL-33 as a prominent component of the inflammatory phenotype (De Boeck et al., 2020). Several studies have demonstrated that CXCL9 is upregulated in glioblastoma tissues and is associated with poor prognosis in glioblastoma patients (Liao et al., 2022).

IL-33 and CXCL9 are secreted by the glioblastoma tissue. However, Cystatin D is primarily highly expressed in serous gland cells in the oral cavity, and FGF21 is mainly secreted by liver cells. Although the secretion of Cystatin D and FGF21 is not directly related to glioblastoma and its associated infiltrating immune cells, recent research has found that both the brain-liver axis and the brain-oral axis can have an impact on the central nervous system. Studies have shown that liver disease may lead to dementia through inflammation, and dysbiosis of the oral microbiota is associated with IDH1 mutant gliomas (Garcia-Martinez and Cordoba, 2012; Wen et al., 2021; Zhao et al., 2021). Therefore, Cystatin D and FGF21 may influence the occurrence of glioblastoma through the brain-oral or brain-liver axes. This provides a promising direction for future research on the impact of the brain-liver axis and the brain-oral axis on glioblastoma.

Our study had a few limitations. Firstly, we applied a significance threshold of *p*-value < 5 × 10^−6^ for the GWAS data on inflammatory proteins due to the limited number of SNPs meeting the more stringent cut-off of *p*-value < 5 × 10^−8^. Second, although our MR-Egger, Weighted Median, Simple mode, and weighted mode estimates did not yield statistically significant results, the IVW method demonstrated higher statistical power than the other MR methods. Moreover, by adhering to the strengthened requirement of a consistent OR direction in our study design, we can consider our findings significant. The third concern pertains to the generalizability of our results across different populations, as all GWAS data were derived from European cohorts; this aspect warrants further investigation. Finally, it is important to acknowledge that various measured and unmeasured confounding factors may have influenced the outcomes of these studies.

## Data Availability

The original contributions presented in the study are included in the article/Supplementary Material, further inquiries can be directed to the corresponding author.
