# Supplementary material for: Causal relationship between inflammatory proteins and glioblastoma: a two-sample bi‑directional mendelian randomization study
Source: Front Genet. 2024 May 9;15:1391921. doi: 10.3389/fgene.2024.1391921 (PMC11111920; doi:10.3389/fgene.2024.1391921)
Supplement: Supplementary file 2 [file Table1.DOCX]

**
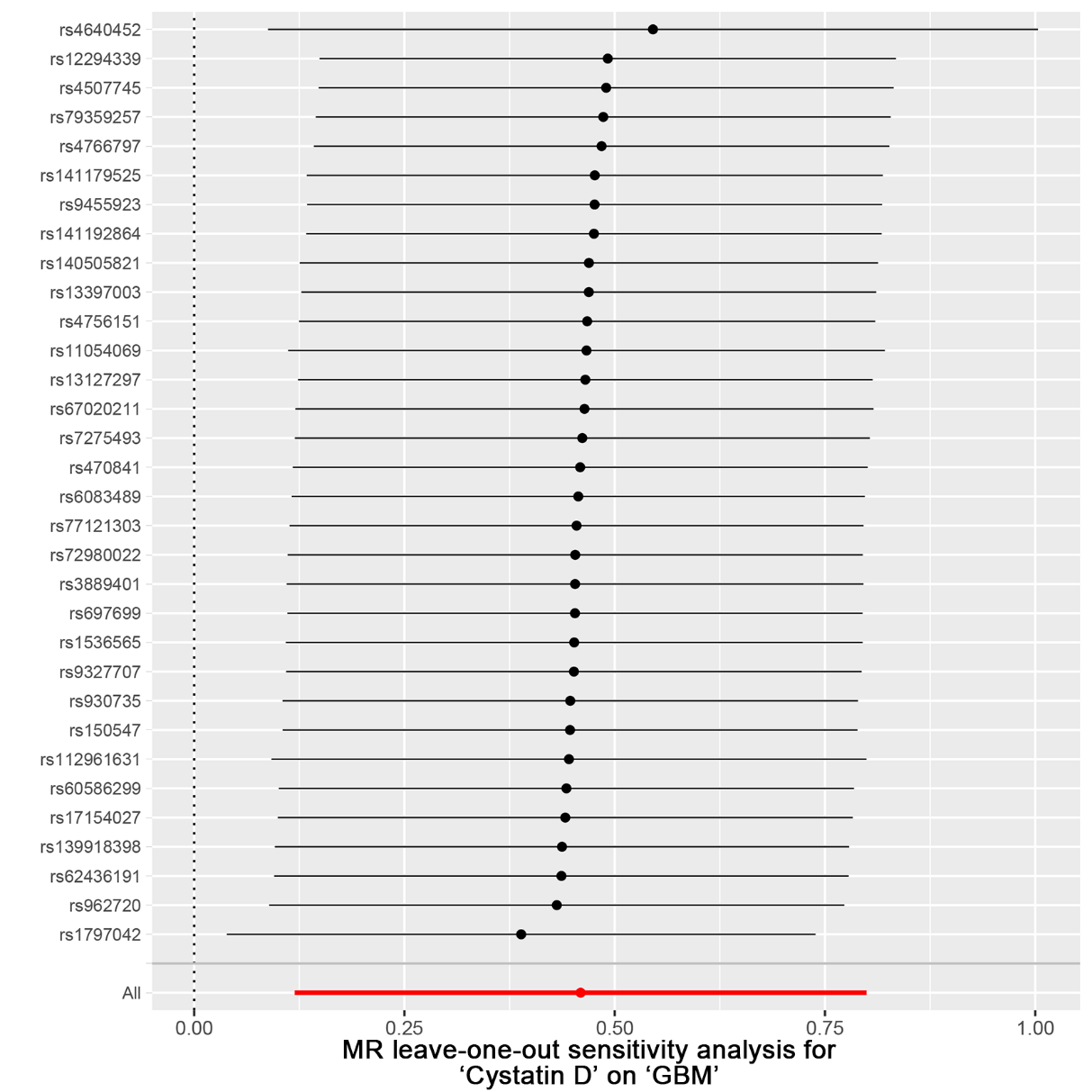
**

**Figure S1** MR leave-one-out sensitivity analysis for Cystatin D on GBM. GBM, Glioblastoma; MR, Mendelian randomization.


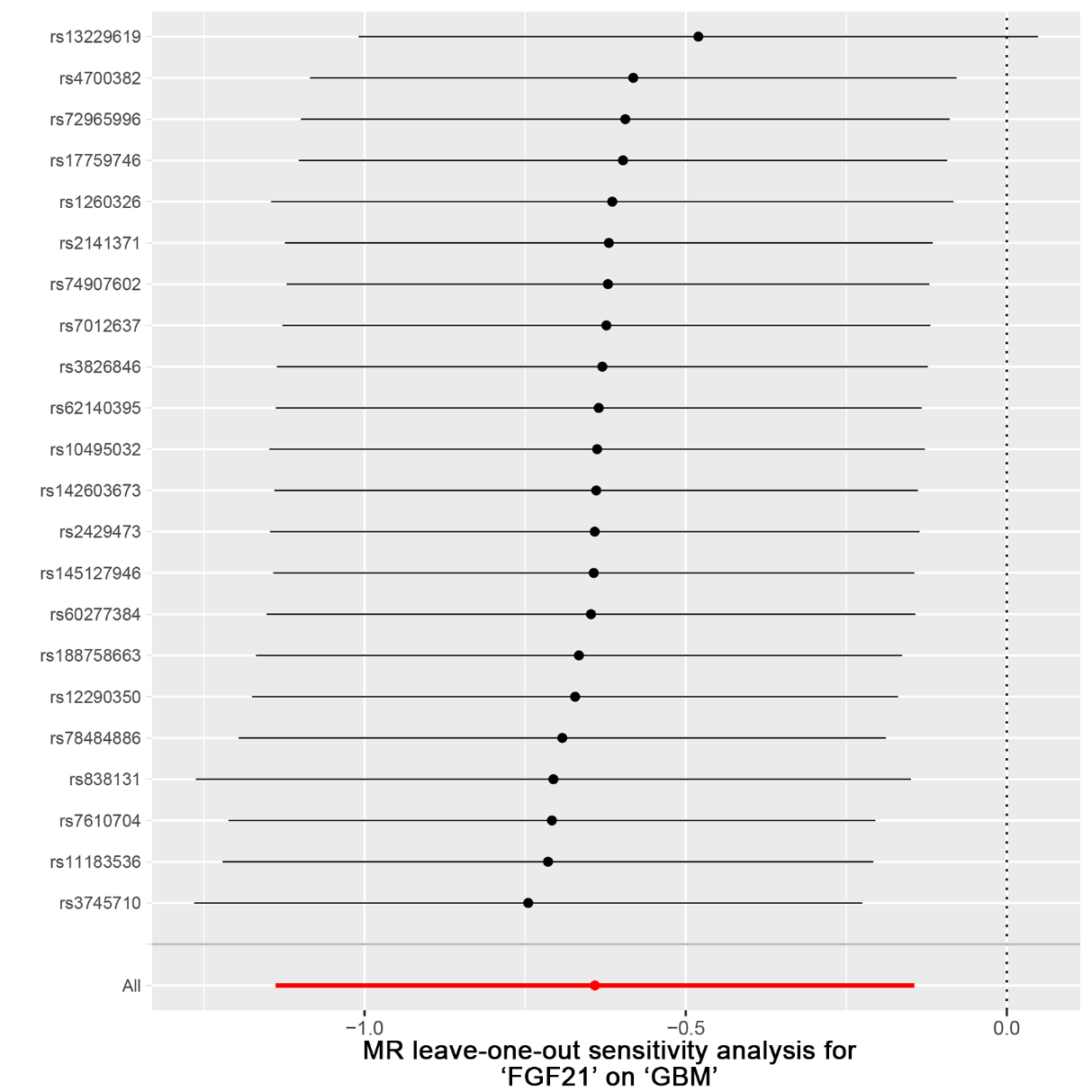


**Figure S2** MR leave-one-out sensitivity analysis for FGF21 on GBM. FGF21, Fibroblast growth factor 21; GBM, Glioblastoma; MR, Mendelian randomization.


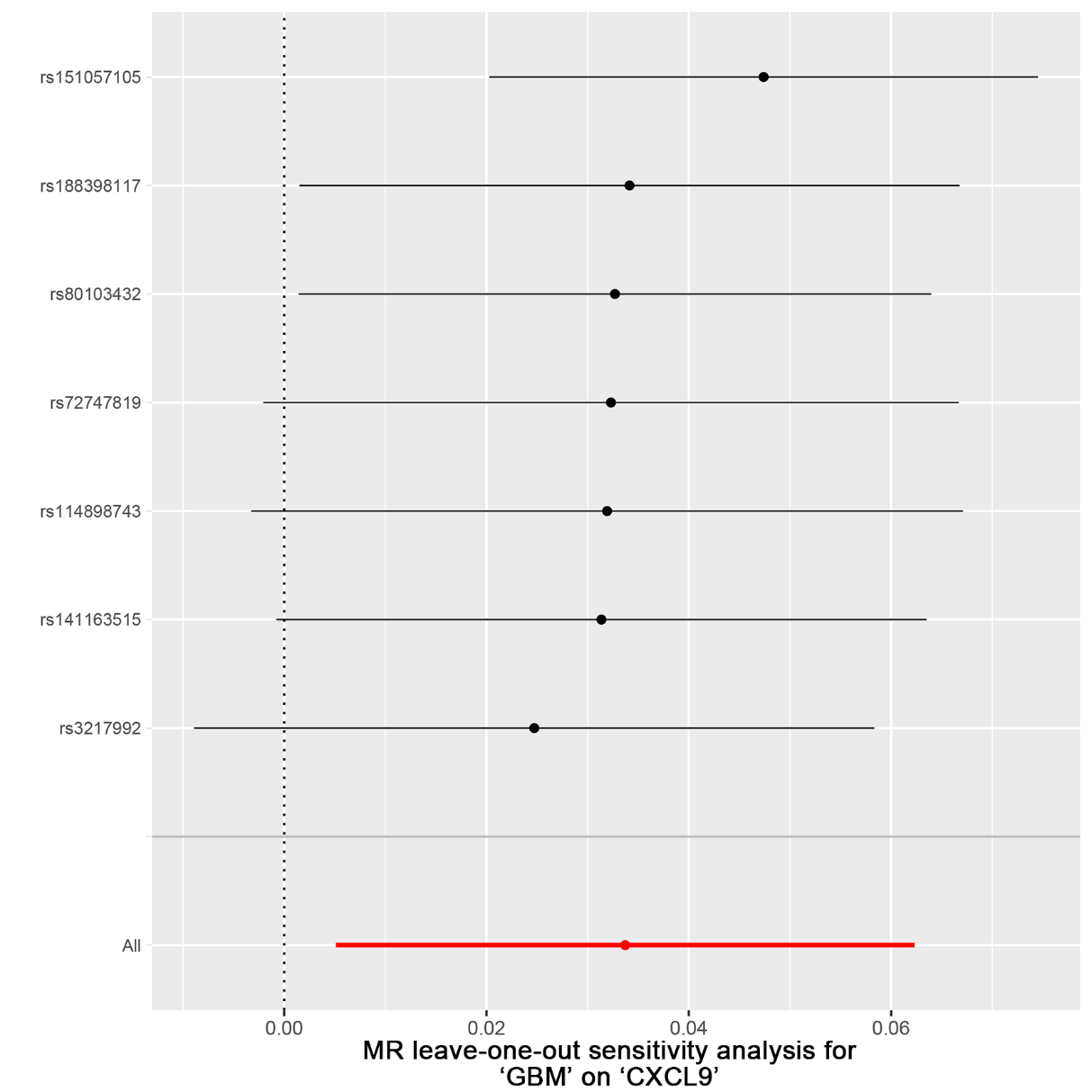


**Figure S3** MR leave-one-out sensitivity analysis for CXCL9 on GBM. CXCL9, C-X-C motif chemokine 9; GBM, Glioblastoma; MR, Mendelian randomization.


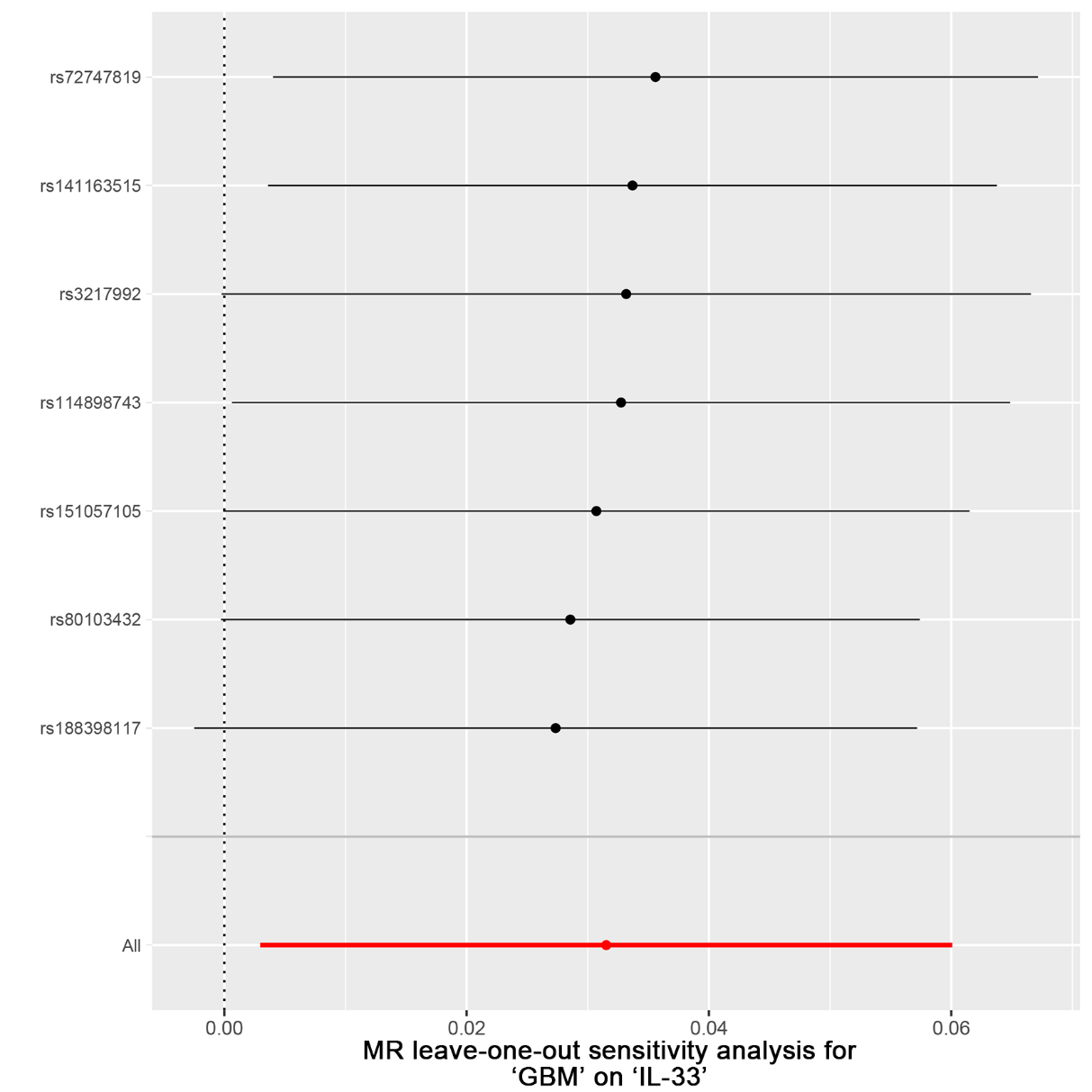


**Figure S4** MR leave-one-out sensitivity analysis for IL-33 on GBM. IL-33, Interleukin-33; GBM, Glioblastoma; MR, Mendelian randomization.


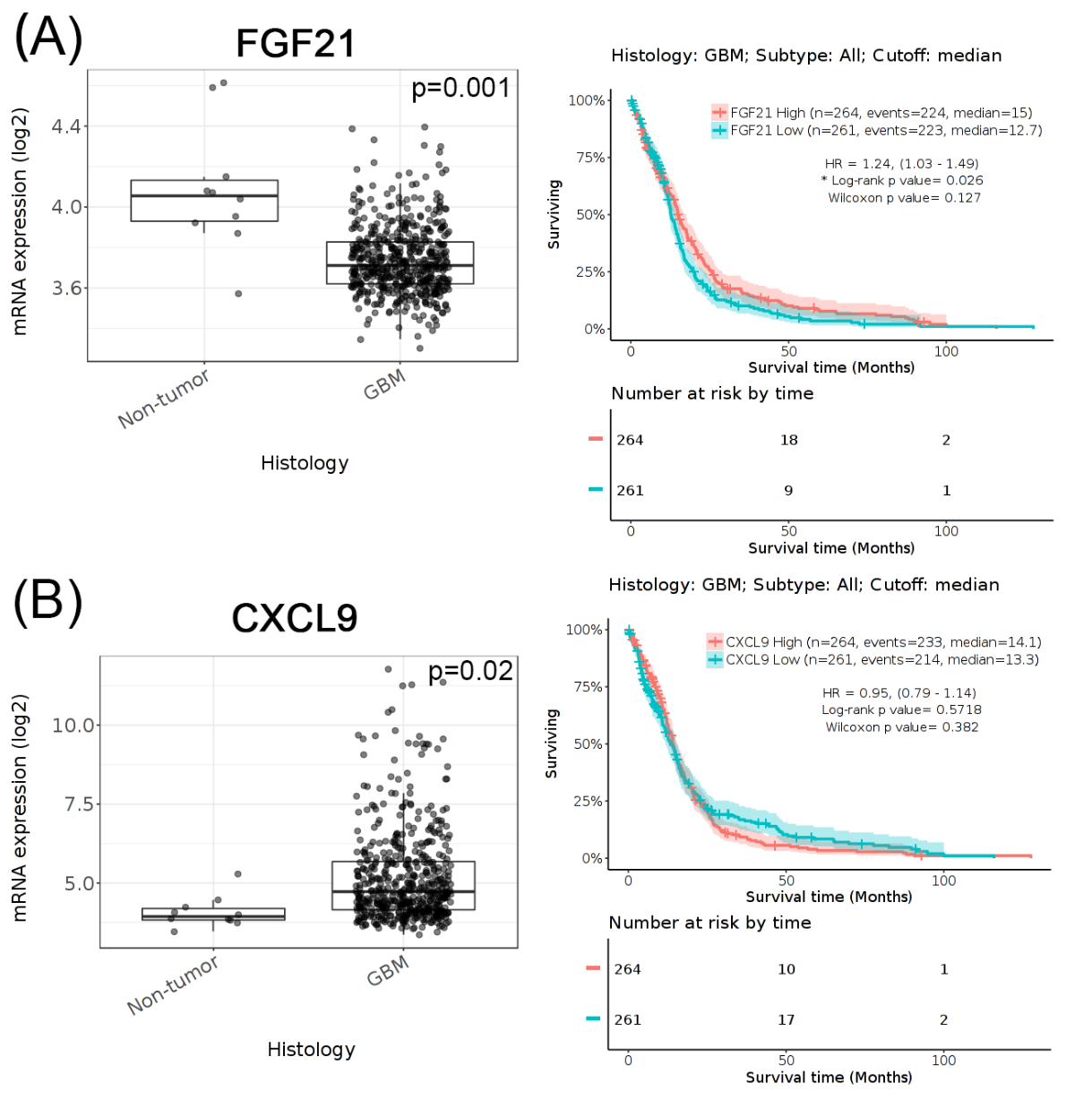


**Figure S5** Transcriptomics analysis of FGF21 and CXCL9. **(A)** Gene expression analysis and Kaplan–Meier survival curves of FGF21 in GBM patients and normal people. **(B)** Gene expression analysis and Kaplan–Meier survival curves of CXCL9 in GBM patients and normal people.


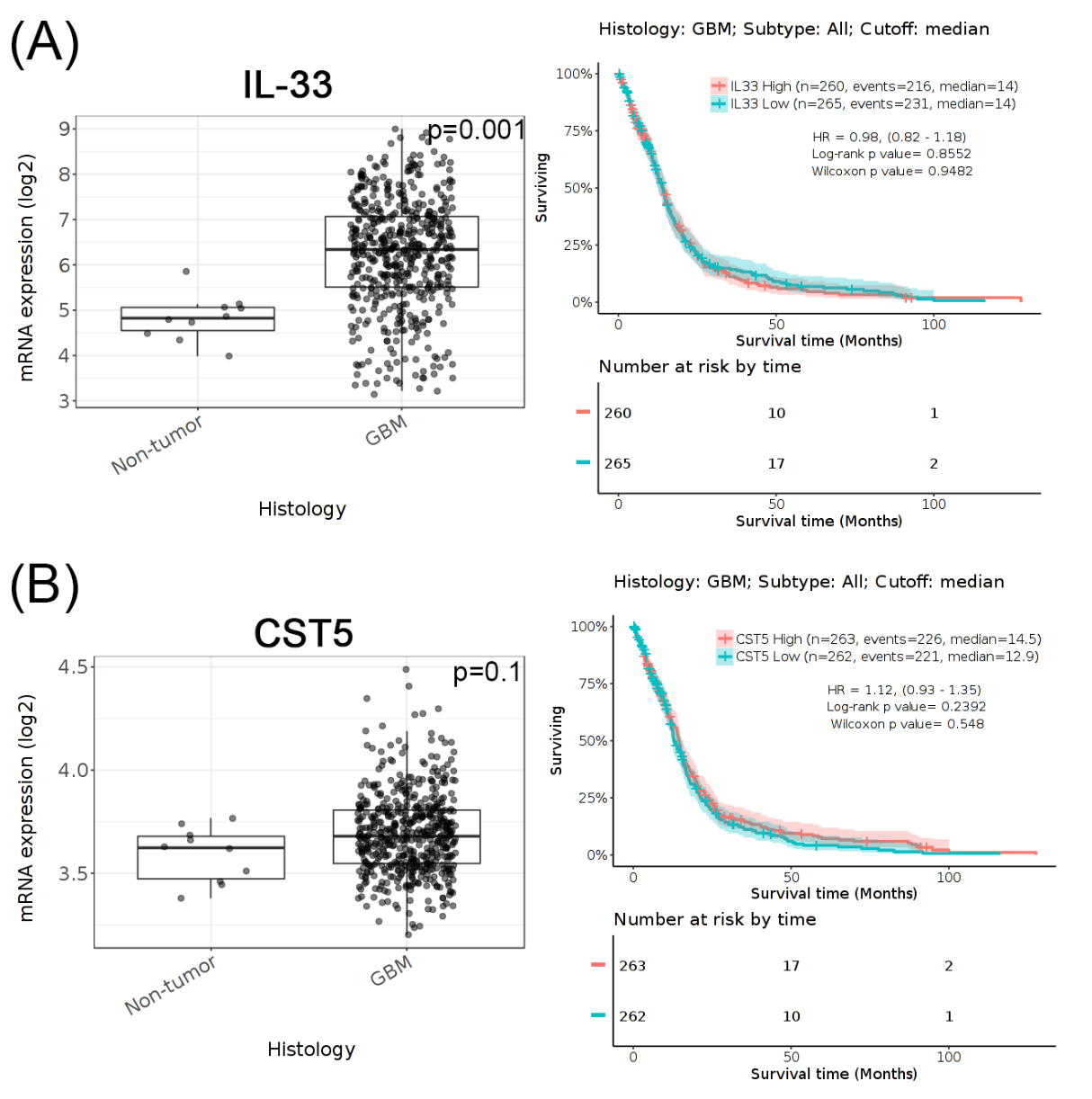


**Figure S6** Transcriptomics analysis of IL-33 and CST5. **(A)** Gene expression analysis and Kaplan–Meier survival curves of IL-33 in GBM patients and normal people. **(B)** Gene expression analysis and Kaplan–Meier survival curves of CST5 in GBM patients and normal people.


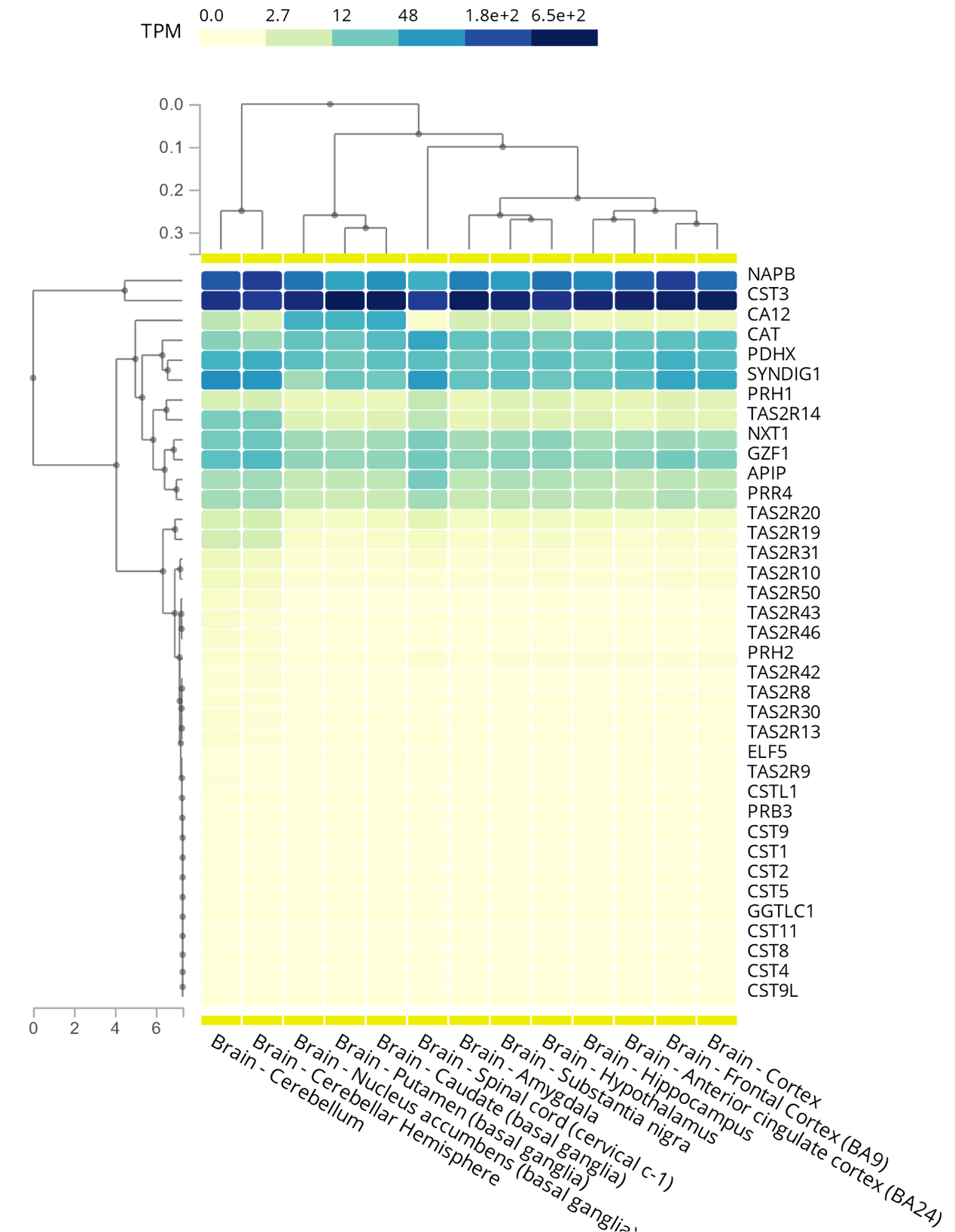


**Figure S7**. Gene expression heatmap of genes associated with SNPs associated with Cystatin D across GTEx v8 brain tissue. SNPs, Single nucleotide polymorphisms.


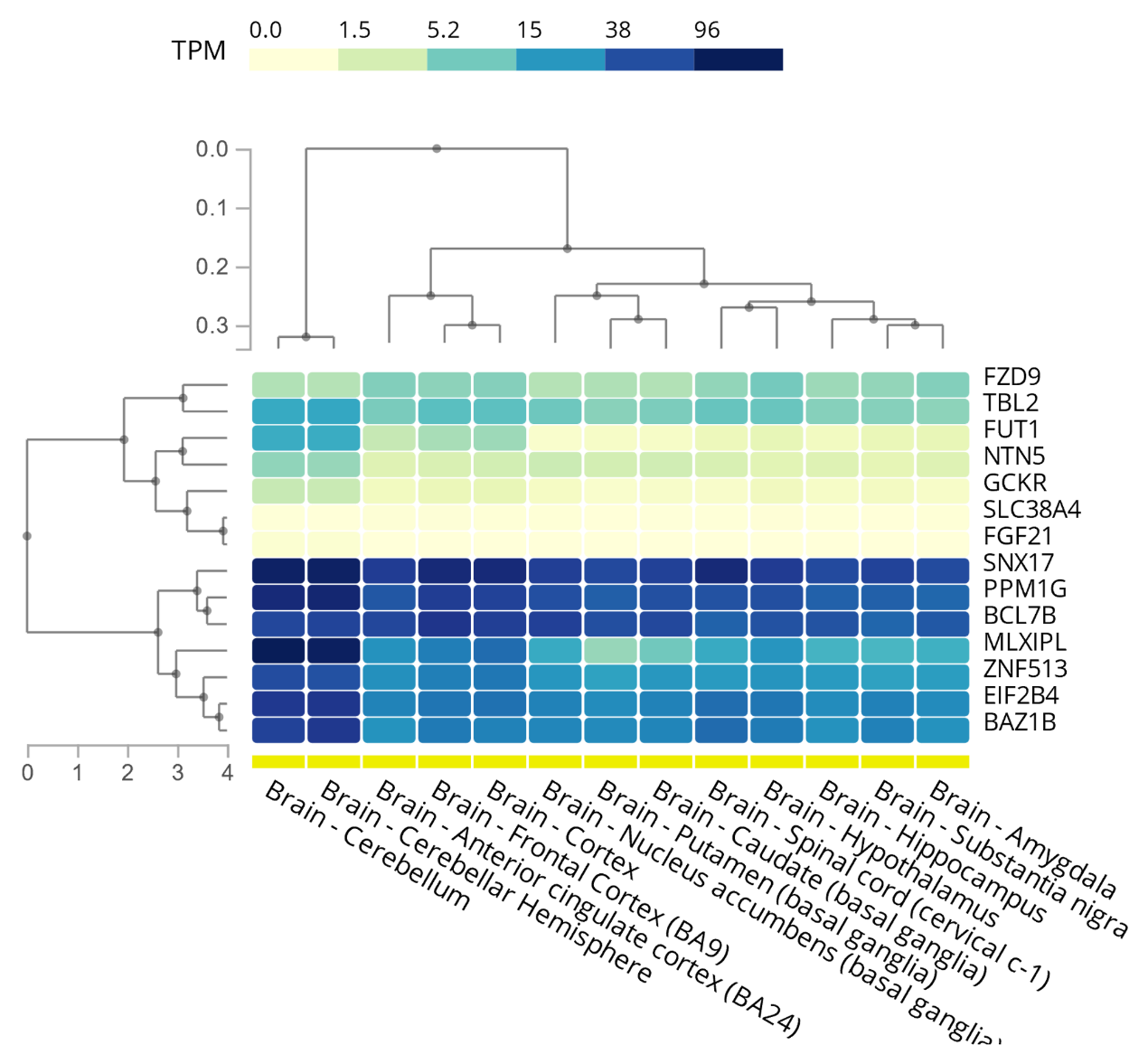


**Figure S8**. Gene expression heatmap of genes associated with SNPs associated with FGF21 across GTEx v8 brain tissue. GTEx v8 brain tissue.


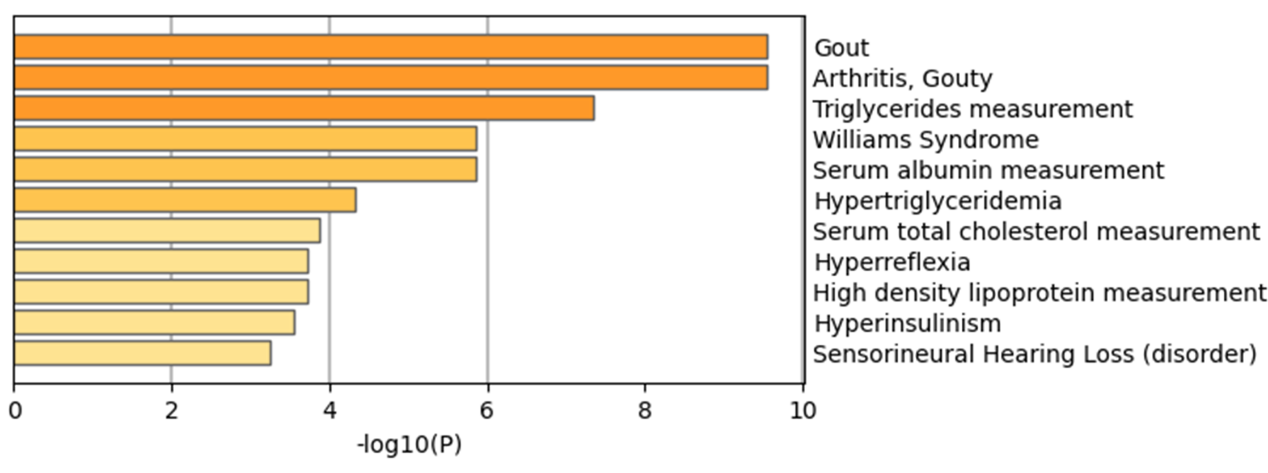


**Figure S9.** Summary of enrichment analysis of FGF21 in DisGeNET


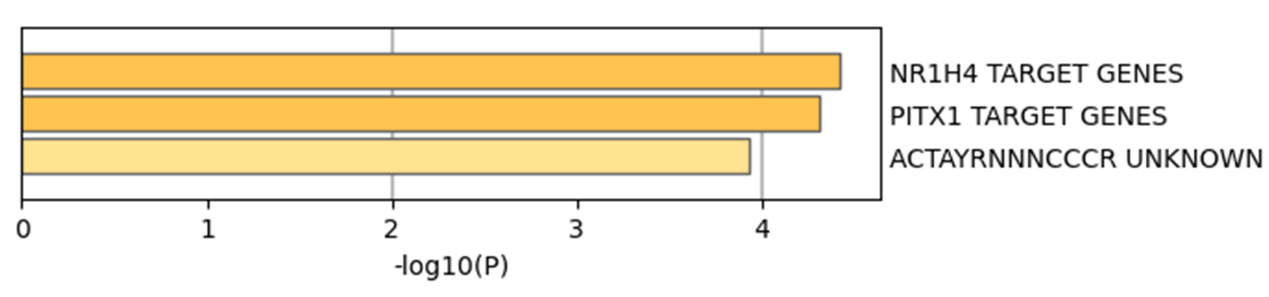


**Figure S10.** Summary of enrichment analysis of FGF21 in transcription factor


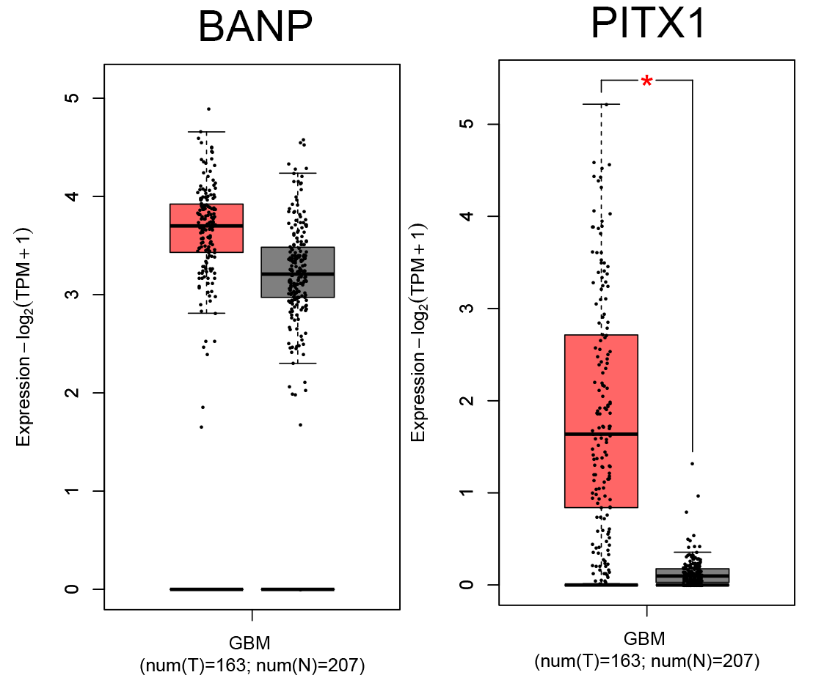


**Figure S11.** Gene expression analysis of BANP and PITX1 in GBM patients and normal people.
